# A standalone approach to utilize telomere length measurement as a surveillance tool in oral leukoplakia

**DOI:** 10.1002/1878-0261.13133

**Published:** 2022-01-07

**Authors:** Jagannath Pal, Yogita Rajput, Shruti Shrivastava, Renuka Gahine, Varsha Mungutwar, Tripti Barardiya, Ankur Chandrakar, Pinaka Pani Ramakrishna, Sovna Shivani Mishra, Hansa Banjara, Vivek Choudhary, Pradeep K. Patra, Masood A. Shammas

**Affiliations:** ^1^ Multi‐Disciplinary Research Unit (MRU) Pt. J.N.M. Medical College Raipur Chhattisgarh India; ^2^ Department of Pathology Govt. Medical College Rajnandgaon Chhattisgarh India; ^3^ Department of Pathology Pt. J.N.M. Medical College Raipur Chhattisgarh India; ^4^ Department of ENT Dr. B.R.A.M. Hospital & Pt. J.N.M. Medical College Raipur Chhattisgarh India; ^5^ Department of Biochemistry Pt. J.N.M. Medical College Raipur Chhattisgarh India; ^6^ Department of Oral Medicine and Radiology Govt. Dental College Raipur Chhattisgarh India; ^7^ Department of Radiotherapy/Regional Cancer Center Dr. B.R.A.M. Hospital & Pt. J.N.M. Medical College Raipur Chhattisgarh India; ^8^ Harvard (Dana Farber) Cancer Institute and VA Boston Healthcare System Boston MA USA

**Keywords:** high‐risk oral habits, oral cancer, oral leukoplakia, relative telomere length

## Abstract

Oral squamous cell carcinoma (OSCC) is often preceded by a white patch on a surface of the mouth, called oral leukoplakia (OL). As accelerated telomere length (TL) shortening in dividing epithelial cells may lead to oncogenic transformation, telomere length measurement could serve as a predictive biomarker in OL. However, due to high variability and lack of a universal reference, there has been a limited translational application. Here, we describe an approach of evaluating TL using paired peripheral blood mononuclear cells (PBMC) as an internal reference and demonstrate its translational relevance. Oral brush biopsy and paired venous blood were collected from 50 male OL patients and 44 male healthy controls (HC). Relative TL was measured by quantitative PCR. TL of each OL or healthy sample was normalized to the paired PBMC sample (TL ratio). In OL patients, the mean TL ratio was significantly smaller not only in the patch but also in distal normal oral tissue, relative to healthy controls without a high‐risk oral habit. Dysplasia was frequently associated with a subgroup that showed a normal TL ratio at the patch but significantly smaller TL ratio at a paired normal distal site. Our data suggest that evaluation of TL attrition using a paired PBMC sample eliminates the requirement of external reference DNA, makes data universally comparable and provides a useful marker to define high‐risk OL groups for follow‐up programs. Larger studies will further validate the approach and its broader application in other premalignant conditions.

## Graphical abstract

Accelerated telomere length (TL) shortening may lead to oncogenic transformation. High variability and lack of universal reference limits translational application of TL‐based surveillance of oral leukoplakia. Here, we demonstrate an approach of using paired peripheral blood mononuclear cells as internal reference. This eliminates the need to use external reference DNA and helps neutralize the impact of age as well as other lifestyle factors. The evaluation of TL in patch and distal normal oral tissue by this simple method could help identify high‐risk category of oral leukoplakia.

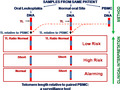

**Abbreviations**
CI: confidence interval
CIN: chromosomal instability
CV: coefficient of variance
HC: healthy control
M: PBMC samples
NH: nonhomogeneous
NOHC: healthy control without oral habit
O: oral samples
OHC: healthy control with oral habit
OL: oral leukoplakia
OLN: paired distal normal oral mucosa in OL patients
OLP: oral leukoplakia patch
OSCC: oral squamous cell carcinoma
PBMC: peripheral blood mononuclear cells
qPCR: quantitative PCR
R: the r statistic (measure of Pearson correlation)
R1: HCT116 cell line
R2: pooled DNA
RR: relative risk
THC: total healthy control
TL: telomere length
TRF: terminal restriction fragment

## 1 Introduction

The high incidence of oral cancer in India could be attributed to oral high‐risk habits which are widespread in the population [1, 2, 3, 4, 5, 6]. The high‐risk oral habits such as chewing tobacco products, smoking and drinking alcohol can be the source of high concentrations of toxic substances for oral mucosa [7, 8]. Chronic exposure of the high‐risk factors frequently leads to a potentially malignant disorder called oral leukoplakia (OL), a white patch‐like oral lesion, diagnosed by exclusion of other known oral diseases that do not carry increased risk for cancer [9, 10]. The final common impact of the toxic chemicals released from these products is oxidative damage within the oral epithelial cells, resulting in DNA damage, chromosomal alterations and carcinogenesis [11, 12].

Telomeres are cap‐like DNA protein structures that protect chromosome ends [13, 14, 15]. In normal somatic cells, telomeres undergo a progressive shortening with cell division, and when TL reaches below a critical limit, the cells undergo replicative senescence [16, 17]. The rate of telomere shortening impacted by a variety of factors including genetics, environment and lifestyle. Accelerated or increased rate of telomere shortening poses a risk for development of cancer [18, 19, 20]. TL shortening seems to occur early in the oncogenesis [21, 22, 23] and certain cancers seem to arise against the background of short TL in surrounding normal tissue [24, 25, 26, 27]. In oral cancer, although most cases show significantly shorter TL, a higher tumor to adjacent normal tissue TL ratio is associated with poor prognosis [26, 28]. This suggests that TL in adjacent or background oral mucosa could be shorter than that in tumor itself. It is suggested that exposure to high‐risk oral habits may contribute to excessive telomere shortening in oral mucosa [29, 30].

Several methods have been developed to measure TL [31, 32, 33, 34, 35, 36]. These methods have unique advantages as well as limitations [37] but cannot be used in clinical practice. This is primarily because TL varies with age, gender, genetics and lifestyle [38]. Moreover, relative TL is usually expressed as a ratio of TL of a reference DNA, which can vary from study to study. A recent study on reproducibility of TL data reported high interlaboratory coefficients of variation for both southern blotting and qPCR [39]. In addition, variation of TL due to ethnicity makes it difficult to analyze these data in mixed multicultural population studies [40]. In some studies, the TL in pathological tissue was compared with that in adjacent healthy tissue [24, 25, 41, 42, 43] to eliminate the impact of other variables. However, this may not be ideal because adjacent tissue could also be abnormal if etiological exposure affected a large area or whole organ [43, 44].

To overcome these issues we proposed that a distant and easily accessible normal tissue may be a better internal control to eliminate impact of variables and batch effect. Recent studies show that in adult tissues the physiological telomere shortening occurs at an equivalent rate and there is a correlation of TL between different tissues from the same individuals [45, 46, 47]. Recently, Finnicum *et al*. (2017) also showed that TL of peripheral blood mononuclear cells (PBMC) and buccal mucosa are significantly correlated [48], suggesting similarities in telomere dynamics between two adult tissue types within an individual. So, it would be quite feasible to express TL of a tissue of interest, relative to TL of easily accessible cells in blood circulation, preferably PBMC. TL of any adult tissue is primarily determined by the TL reserve of its progenitor cell [45]. Although PBMC can pass through oral mucosa, their progenitor cell pool is located away from oral mucosa. So, whereas oral mucosa is exposed to oral high‐risk factors, the progenitor cells of PBMC, because of their distal location, are less exposed to these factors. Moreover, a small fraction of the PBMC passing through oral mucosa may overall be less impacted by toxic exposure of high‐risk habits than the localized cells of oral mucosa. Therefore, local pathological TL attrition in oral mucosa could be evaluated, using PBMC as reference. This should eliminate/minimize the impact of the personalized variables mentioned above and allow utilization of TL as a clinical marker. Additionally, the use of paired PBMC samples will also eliminate the need of an external reference DNA, which may vary from laboratory to laboratory and from study to study.

We, therefore, explored the feasibility of utilizing the ratio of TL of OL and paired PBMC sample as a clinical marker for evaluation of OL.

## 2 Materials and methods

### 2.1 Ethical statement

This study was carried out at Multi‐Disciplinary Research Unit (MRU), Pt. Jawaharlal Nehru Memorial Medical College, Raipur, Chhattisgarh, India, following approval of the protocol by the institutional ethical committee. All procedures were carried out in accordance with the Declaration of Helsinki. Human samples were collected following informed written consent from the study subjects.

### 2.2 Study participants

Patients who attended ENT and dental clinic with oral lesions, had clinical diagnosis of leukoplakia and were willing to participate in the study, were referred to MRU for enrollment. Normal course of clinical management of the patients was not hampered by the study protocol. Immunocompromised patients and those undertaking any therapy for the lesion or with history of other chronic diseases, bleeding tendency or any acute illness were excluded from the study.

### 2.3 Sample collection and processing

#### 2.3.1 Brush biopsy and blood collection

Prior to oral brush biopsy, study subjects were asked to rinse their mouths twice thoroughly with normal saline. For each patient, brush biopsy was performed using a small‐headed baby toothbrush on the leukoplakia (OLP) and on a normal site (OLN), preferably opposite the lesion. In healthy controls, buccal samples were collected from cheeks. Cells were dislodged into PBS, slides were prepared for cytological assessment and remaining cells stored at −80 °C in 10% DMSO. Blood was collected by venepuncture in EDTA vials. Clinically high‐risk/suspicious OL or brush biopsy samples showing dysplasia were confirmed by punch biopsy.

#### 2.3.2 Isolation of PBMC

Isolation of PBMC was done using Granulosep GSM and Hisep LSM (HiMedia, Mumbai, India) as per the manufacturer’s protocols. Cell pellet was resuspended in 10% DMSO in RPMI 1640 media and stored in aliquots at −80 °C till further use.

#### 2.3.3 DNA isolation and quantification

All paired oral brush biopsy samples and PBMC were processed simultaneously. DNA isolation was done by DNeasy blood and tissue kit (Qiagen, Hilden, Germany) using the manufacturer’s protocol with little modification. Proteinase K treatment was carried out overnight at 56 °C. For elution, the column was kept with elution buffer at room temperature for 2 h before centrifugation. DNA quantization was performed by Quant‐iT™ PicoGreen™ dsDNA Assay Kit (Invitrogen, Eugene, OR, USA) as per the manufacturer’s protocol using Multimode Microplate Reader (TECAN, Mannedorf, Switzerland).

### 2.4 Relative telomere length assay by qPCR

Telomere length (TL) measurement was performed using the quantitative PCR method (qPCR) as described by O’Callaghan and Fenech [35] with slight modification. Instead of absolute telomere length as described by O’Callaghan *et al*., we measured relative telomere length. Genomic DNA samples from all patients were normalized to 1 ng·µL^−1^ and variation < ± 0.05 ng·µL^−1^ DNA was tolerated. For each qPCR reaction, 4‐ng genomic DNA was used. A symmetry was maintained on the PCR plate in positioning of the telomere and single copy gene amplification reaction for each sample so that any possible positional effect of the wells could be nullified.

The T/S (telomere repeat amplification/single copy gene amplification) ratio for each sample and reference DNA was calculated using the formula 2^−ΔCt^. For measuring relative TL of buccal epithelium, the T/S ratio of buccal cells (O) was divided by the T/S ratio of paired PBMC (M) or reference DNA (HCT116 (R1) or Cocktail DNA (R2) as appropriate. CV of rTL ratio between the plates was determined by running HCT116 cell line DNA and one of the two batches of cocktail of 10 brush biopsy samples from a healthy control pair in four different qPCR plates on different days for each cocktail. CV was 4.5% and 8% for the two cocktails, respectively.

### 2.5 Statistical analysis

Mean, standard deviation, median and Pearson correlation were performed. Kolmogorov–Smirnov Test of Normality, comparison of mean (*t*‐test), significance of Pearson’s correlation, significance of association (Fisher exact probability due to small sample size) and relative risk were analyzed using the following web‐based software: medcalc (htmlhttps://www.medcalc.org/calc/odds_ratio.php) and social science statistics (https://www.socscistatistics.com/tutorials/ttest/default.aspx). *P*‐values ≤ 0.05 were considered statistically significant.

## 3 Results

### 3.1 Demographic profile of healthy controls and leukoplakia patients

Since the number of female OL patients was very low and females, especially in India, behave differently in terms of high‐risk oral habits (as reported by us previously [6]), in this pilot study we only evaluated male OL patients and compared their relative telomere lengths with those of a male healthy control group. Samples with low quantity or poor quality of DNA were removed and 50 OL and 44 healthy control samples were processed for TL analysis. Mean ages of OL patients and healthy controls were 42.24 ± 12.08 years (range 20–67 years) and 36.36 ± 13.47 years (range 20–69 years), respectively (Supporting Information Table S1).

### 3.2 Profile of high‐risk habits

An oral habit was considered to be high risk if carried out regularly at least for 2 years consecutively. In the case of multiple oral habits, the duration of the longest regular habit was considered. High‐risk oral habits included smoking tobacco, chewing raw tobacco (khaini or cut tobacco leaves mixed with slaked lime), chewing gutkha (Areca nut, slaked lime, catechu and tobacco with flavorings and sweeteners) and drinking alcohol. Of 44 healthy donors, 25 had no recognizable high‐risk habit, whereas 19 had one or different combinations of more than one high‐risk oral habits. Mean duration of the habit in the high‐risk healthy control group was 10.13 ± 8.34 years. Among the OL patients, 47 had high‐risk oral habit/s and three had no high‐risk oral habit. Mean duration of the habit in the high‐risk OL group was 15.47 ± 11.40 years. High‐risk oral habits in OL patients existed in different combinations. Detail duration and profile of high‐risk habits in healthy control and OL patients are given in Supporting Information Tables S2 and S3, respectively.

### 3.3 Evaluation of relative TL of oral mucosa relative to paired PBMC sample in healthy controls

**Fig. 1:**
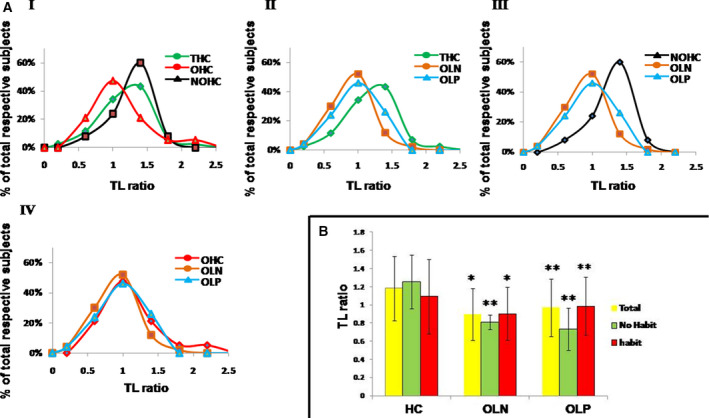
Frequency and mean distribution of telomere length ratio in oral mucosa of healthy control and oral leukoplakia patients. (A) Comparison of frequency distribution of telomere length (TL) ratio of oral leukoplakia (OL) patients with healthy controls (HC). (I) Comparative distribution of all healthy controls (THC; *n* = 44), healthy controls without oral habit (NOHC; *n* = 25) and healthy controls with high‐risk oral habit (OHC; *n* = 19). (II) Comparative distribution of paired distal normal mucosa in OL patients (OLN; *n* = 50), oral patch in OL patients (OLP; *n* = 50) and THC (*n* = 44). (III) Comparative distribution of OLN (*n* = 50), oral patch in OL patients (OLP; *n* = 50) and NOHC (*n* = 25). (IV) Comparative distribution of OLN (*n* = 50), OLP (*n* = 50) and OHC (*n* = 19). (B) Bar graph showing mean of TL ratio in OL and HC groups. The bar graph represents the mean TL ratio in HC (total, *n* = 44; no habit, *n* = 25; with habit, *n* = 19), OLN (total, *n* = 50; no habit, *n* = 3; with habit, *n* = 47) and OLP (total, *n* = 50; no habit, *n* = 3; with habit, *n* = 47). Color code: yellow, green and red colors represent total, no habit and with habit, respectively, as indicated. Error bar indicates standard deviation. Significance of mean difference (*t*‐test) *P* < 0.5 is considered statistically significant; *P indicates significant when compared with all groups of healthy controls (total, no habit, habit) and **P indicates significant when compared with total and no‐habit healthy control.

The purpose of this study was to investigate whether assessment of relative TL of oral mucosa using paired PBMC samples from the same subject (as internal reference) could serve as a prognostic tool. We first evaluated whether this approach can separate two groups of healthy individuals (HC), i.e. individuals with high‐risk habits and those without these habits. We initially used a small number of HC samples, seven with and nine without high‐risk oral habits, and determined relative TL using paired PBMC sample as internal reference. To compare this strategy with the existing method of using external reference DNA, we also assessed relative TL in these samples using two external controls (i.e. DNA from HCT116 cell line and pooled DNA from 10 brush biopsy samples of HC). T/S ratios of oral samples (O) were divided by the T/S ratio of the corresponding paired PBMC samples (M), HCT116 cell line (R1) or the pooled DNA (R2), thus expressing relative TL as O/M, O/R1 or O/R2, respectively. As expected, we observed TL attrition in the high‐risk group when relative TL was expressed as O/M (*P* = 0.056), whereas no differences were observed when external reference DNA values were used (*P* = 0.746 and 0.342, respectively) (Supporting Information Fig. S1A,B,C). Based on these results, we determined the relative TL of oral mucosa in all healthy individuals using paired PBMC samples as reference (O/M) and this parameter is referred to as the ‘TL ratio’ in this manuscript. Frequency distribution curves of TL ratios of healthy controls (with and without high‐risk habits) are shown in Fig. 1AI. Overall, the mean TL ratio of all healthy subjects was 1.18 ± 0.36 (95% CI 1.076–1.292). The mean TL ratios in healthy male subjects without and with high‐risk habits were 1.25 ± 0.30 (95% CI 1.130–1.375) and 1.09 ± 0.41 (95% CI 0.897–1.292), respectively (Table 1, Fig. 1B). Median values of both the groups were 1.297 and 1.002, respectively. The TL ratios did not correlate with age (Table 1) and were normally distributed (Supporting Information Table S4). We did not observe any significant difference of mean between high‐risk and no high‐risk oral habit groups (*P* = 0.14; Table 1). In our high‐risk habit HC group, most of the habits existed in combinations and due to small sample size, and thus we could not analyze the impact of individual high‐risk habits. However, when we determined TL ratio in HC individuals having any of the high‐risk habits, irrespective of the presence of other habits, we only observed a significant small TL ratio (0.93 ± 0.38, *P* = 0.02) in the group having the habit of chewing khaini (Table 1).

**Table 1:** Mean telomere length ratio in healthy control group in relation to oral high‐risk habits. TL, telomere length; HC, healthy control; Habit, oral high‐risk habit; No habit, no oral high‐risk habit; n, number of subjects.

HC	*n*	TL ratio in oral tissue of HC		Correlation with Age vs. TL ratio R (*P*)
		Mean ± SD	*P* (vs. No habit)	
Total	44	1.18 ± 0.36	‐	–0.069 (0.747)
No habit	25	1.25 ± 0.30	‐	
Habit	19	1.09 ± 0.41	0.14	0.210 (0.388)
Smoking	6	1.23 ± 0.51	0.88	
Chewing raw tobacco (khaini)	7	0.93 ± 0.38	0.02*	
Chewing gutkha	10	1.27 ± 0.41	0.89	
Drinking alcohol	9	1.18 ± 0.46	0.59	

*P, *P*‐value is significant at the significance level *P* ≤ 0.05; R: the *r* statistic (measure of Pearson correlation).

Based on these observations, we determined the lower cut‐off for the TL ratio in healthy male individuals. For males, the TL ratio of 0.765 was set as a threshold (95th percentile of no high‐risk habit healthy individuals; one‐sided Z‐score 1.645); a TL ratio below this could be considered to represent a significantly shorter TL.

### 3.4 Analysis of TL ratio in oral leukoplakia

TL ratio (O/M) was assessed in 50 oral leukoplakia samples. Age composition is described in Table S1. Oral leukoplakia predominantly occurs in males with high‐risk oral habits. Consistently, of 50 male patients, 47 (94%) had recognizable high‐risk oral habits. For each patient, oral brush biopsy samples were collected from an oral leukoplakia patch (OLP) as well from a distal visually normal site (OLN), preferably from an opposite anatomical site in the oral cavity. All the values of TL ratio in OL patients were normally distributed (Table S4).

Frequency distribution curves of TL ratio of the OL patient samples compared with corresponding healthy controls (HC) are shown in Fig. 1A. Peak TL ratio distributions of both OLN and OLP were clearly in the shorter range of TL compared with corresponding HC samples without any habit. However, TL ratio distribution of HC with high‐risk oral habits was very close to the distribution of OL samples (Fig. 1AIII, IV). Overall, mean TL ratios of OLN and OLP in all OL patients were 0.90 ± 0.28 and 0.97 ± 0.32, respectively. The same OL patients with high‐risk habit were 0.90 ± 0.29 and 0.99 ± 0.32, respectively. In patients with no high‐risk habit, mean TL ratios of OLN and OLP were 0.81 ± 0.08 and 0.73 ± 0.23, respectively (Table 2, Fig. 1B). Though the mean TL ratio in OLP was slightly greater than OLN, it was not significantly different. However, a statistically significant positive correlation was observed between OLP and OLN (0.389; *P* = 0.005) (Supporting Information Table S5). As in healthy control groups, we did not observe any correlation of TL ratio with age in OL patients (Table S5), suggesting the parameter is age‐neutral.

**Table 2:** Telomere length ratio in OL patient subgroups. TL, telomere length; Habit, oral high‐risk habit; No habit, no oral high‐risk habit; OLN, paired distal normal mucosa in OL patients; OLP, oral patch in OL patients; n, number of subjects.

OL patients	*n*	TL ratio OL			
		OLN		OLP	
		Mean ± SD	*P*	Mean ± SD	*P*
Habit/no habit			(vs no habit HC)		(vs no habit HC)
Total	50	0.90 ± 0.28	< 0.0001*	0.97 ± 0.32	0.0005*
Habit	47	0.90 ± 0.29	< 0.0001*	0.99 ± 0.32	0.0012*
Smoking	18	0.99 ± 0.35	0.0110*	1.00 ± 0.40	0.0217*
Chewing raw tobacco (Khaini)	21	0.91 ± 0.26	0.0002*	0.93 ± 0.32	0.0009*
Chewing gutkha	26	0.93 ± 0.27	0.0002*	1.01 ± 0.32	0.0071*
Drinking alcohol	20	0.96 ± 0.27	0.0013*	1.01 ± 0.320.	0.0115*
No habit	3	0.81 ± 0.08	0.018*	0.73 ± 0.23	0.0071*
Clinical high/low risk			(High vs low risk)		(High vs low risk)
Homogeneous	35	0.91 ± 0.30	0.596	0.99 ± 0.34	0.467
Nonhomogeneous	15	0.86 ± 0.26		0.92 ± 0.26	
Pathological high/low risk			(High vs low risk)		(High vs low risk)
No dysplasia	46	0.92 ± 0.28	0.046*	0.98 ± 0.33	0.404
Dysplasia/carcinoma	4	0.63 ± 0.06		0.84 ± 0.13	
Clinico‐pathological high/low risk (combined)			(High vs low risk)		(High vs low risk)
Homogeneous without dysplasia	32	0.94 ± 0.30	0.208	1.01 ± 0.35	0.291
Nonhomogeneous and or dysplasia	18	0.82 ± 0.25		0.91 ± 0.25	

*P, *P*‐value is significant at the significance level *P* ≤ 0.05.

The mean TL ratios of OLN and OLP in all groups of OL patients (total, high‐risk and no high‐risk oral habit groups) were significantly lower compared with the HC group having no high‐risk oral habit (Table 2). When the means of the TL ratio in OLN and OLP were compared with the HC group with high‐risk oral habits, although both the OLN and OLP (in all the subgroups of OL patients) showed a slightly lower TL ratio, the significance was observed only with OLN, not with OLP (Supporting Information Table S6). Among the high‐risk oral habits, chewing khaini was associated with the smallest TL ratio in both OLN and OLP (0.91 ± 0.26 and 0.93 ± 0.32, respectively), whereas smoking tobacco had the least effect (Table 2). However, no significant correlation was observed between TL ratio and duration of the habits (Table S2).

### 3.5 Classification of OL based on TL ratio

Using 0.76 as the lower limit of normal TL ratio of oral epithelium, we identified 22 of 50 (44%) cases of OL patients who had significantly smaller relative TL ratio, at least in one oral sample (OLP/OLN). Based on the established TL ratio threshold value, OL patients were categorized into four subgroups: (A) OLN normal – OLP smaller; (B) OLN smaller – OLP normal; (C) OLN smaller – OLP smaller; (D) OLN normal – OLP normal. The respective percentages of OL patients in the groups A, B, C and D were 10% (5/50), 20% (10/50), 14% (7/50) and 56% (28/50), respectively (Table 3).

**Table 3:** Clinicopathological association with the telomere length ratio‐based subgrouping of OL. N, OLN; P, OLP; G, Number in the corresponding group of OL (A/B/C/D); NH, nonhomogeneous OL; *n*, number of nonhomogeneous OL or dysplasia cases as indicated.

OL Group: (normal/small TL ratio in paired N and P)	% of total OL (50): % (G)	NH: % of G(*n*) p (vs. D)		Dysplasia/carcinoma: % of G (*n*) p (vs. D)		Clinicopathological high‐risk OL (NH+Dysplasia/carcinoma) % of G(*n*) p (vs A + D)
A (N:normal, P:small)	10 (5)	20 (1) *P* = 1.00		0 (0) *P* = 0.559		(B + C):59 (10) *P* = 0.030* RR:2.42 (95%CI:1.18‐5.00, *P* = 0.016*)
B (N:small, P:normal)	20 (10)	30 (3) *P* = 1.00	(B + C): 41 (7) *P* = 0.327	30 (3) *P* = 0.014*	(B + C): 24 (4) *P* = 0.016*	
C (N:small, P:small)	14 (7)	57 (4) *P* = 0.171		14 (1) *P* = 0.200		(A + D): 24(8)
D (N:normal, P:normal)	56 (28)	25 (7)		0 (0)		
Total	100 (50)					

*P, *P*‐value is significant at the significance level *P* ≤ 0.05 (*P*‐values determined by Fisher exact probability due to small sample size); RR: relative risk.

### 3.6 Clinicopathological association with the TL‐based classification of OL

As nonhomogeneous OL (NH) is one of the high‐risk clinical presentations of OL for malignant transformation [49], the frequency of NH was evaluated in TL‐based OL subgroups. NH was defined as predominantly white or white and red (speckled), with irregularly flat, nodular or verrucous oral lesions [50]. We identified 15 NH out of the 50 OL samples. Mean TL ratio in homogeneous (H) OL were higher than nonhomogeneous (NH) OL in both OLN and OLP (Table 2). The percentage distribution of NH in different subgroups of OL (A, B, C, D; Table 3) was the highest in group C. In this study, only four of 50 male OL patients (8%) were identified with oral dysplasia or early‐stage carcinoma (two dysplasia and two carcinoma). We analyzed them as being in the category of bad pathological outcome. The mean TL ratios in no dysplasia/carcinoma and dysplasia/carcinoma groups were 0.92 ± 0.28 and 0.63 ± 0.06, respectively, for OLN. For OLP, ratios were 0.98 ± 0.33 and 0.84 ± 0.13, respectively (Table 2). We observed the occurrence of all dysplasia/carcinoma samples only in groups B and C. Overall in B and C, 24% of samples were cases of dysplasia/carcinoma (Table 3). Corresponding relative risk (RR) and *P*‐values for association of dysplasia/carcinoma with group B and C compared with group D were 18.45 (95% CI 1.04–329.02), *P* = 0.014, and 10.88 (95% CI 0.488–242.24), *P* = 0.20, respectively. Due to small sample size, the test of significance (*P*) was determined by the Fisher exact probability test. For combined B and C, the value was 14.50 (95% CI 0.829–253.697), *P* = 0.016.

As the clinicopathological presentations of nonhomogeneous OL, dysplasia and carcinoma all carry a high risk of transformation into invasive malignancy, we combined all these presentations into one high‐risk category. With 18 of 50 OL cases in this high‐risk category, the TL ratio at OLN site was significantly lower in 55.5% (10/18) of cases, but only in 21.9% (7/32) of cases with low‐risk OL (homogeneous OL without any dysplasia/carcinoma). Relative risk of association of high‐risk OL at OLN with shorter telomeres (B + C) was 2.43 times more likely than OLN with long telomeres (A + D) (RR: 2.43; 95% CI 1.18–5.00; *P* = 0.0160) (Table 3). These observations indicate that the high‐risk subgroups of OL are more frequently associated with shorter TL in distal normal oral mucosa (OLN), irrespective of TL status of OL patch.

## 4 Discussion

In this study, we demonstrate that the assessment of TL in a lesion or tissue using a paired PBMC sample from the same patient can eliminate the limitations related to utilization of TL as a clinical marker. Using this approach, we were able to separate OL patients into four groups based on their TL in OL patches and corresponding distal normal sites, and showed the clinicopathological relevance. The approach was able to detect the pathological changes in TL in oral epithelium even in apparently healthy individuals with high‐risk habits. Moreover, the observed changes in TL ratios could not be attributed to age differences as they did not show any correlation with age. This is because normalization with paired PBMC sample eliminates the impact of age and other personal factors.

Very few studies have evaluated the direct impact of high‐risk oral habits on TL of healthy oral mucosa. To our knowledge, this is the first report of the impact of smokeless tobacco (khaini) on TL of oral mucosa. However, the sample size in this study was small and there is also the possibility that the impact on telomeres was due to the interaction between chewing raw tobacco with other habit/s. It is not clear why chewing gutkha, which is also a class of smokeless tobacco, did not show any impact on TL in healthy individuals. It may be possible that gutkha, being a processed product, has less impact than raw tobacco because of differences in toxic chemicals or their concentrations.

Although OL patches develop on a focal area, prior exposure to high‐risk factors always involves larger areas of oral mucosa. So, to understand the genomic pathology and disease outcome, it is important to evaluate the patch as well as apparently healthy tissue at distant site. Therefore, unlike previous studies, we sampled the patch area as well as clinically normal mucosa from an anatomically opposite side. Using this approach, we demonstrated that telomere shortening occurs not only in the patch but also in apparently healthy oral mucosa at an anatomically distant site. Overall, the TL ratio in OL patients was smaller at the normal site (OLN) than at the paired patch (OLP). However, a larger sample size may be needed to observe a statistical difference. Although the TL ratio in the no‐habit OL group was smaller than that in high‐risk habit OL group, we are cautious about drawing concluions based on this because of the small sample size of no‐habit group. Interestingly, in the absence of high‐risk oral habits, OL is more prone to carcinogenic transformation [51]. It is possible that in the absence of high‐risk oral habits, other factor/s (e.g. HPV infection) are involved in the etiology of OL.

Paradoxically, TL ratio did not correlate with the duration of high‐risk habits in either the HC or the OL groups. We defined as high risk, a habit of at least 2 years. It is possible that 2 years is long enough to initiate damage leading to sustainable telomere attrition. Consistent with high‐risk habit HC data, OL patients with the habit of chewing khaini also had the shortest TL in both OLN and OLP. However, in OL patients, the habit of chewing gutkha, another form of smokeless tobacco, also had the second strongest impact on TL in OLN, whereas smoking had the least impact (Table 2). Moreover, in OL patients with single habit, chewing khaini was associated with the shortest TL in OLN (Supporting Information Table S7). These data are consistent with the report that smokeless (raw) tobacco causes higher oxidative DNA damage and is more carcinogenic than smoking tobacco or drinking alcohol [52, 53, 54]. Telomeres in OLP samples of all the habit groups were little longer and less consistent compared with those in OLN. This could be due to a varying degree of reactivation of TL maintaining mechanism/s in the patch area against a background of shorter telomeres in oral mucosa.

In our study, only four of 50 OL patients (8%) were identified as having dysplasia/carcinoma. This is consistent with a previous study in which screening of 2920 OL cases revealed that 8.6% were dysplasia, 0.72% malignant and 9.3% either dysplasia or malignant [55]. Based on combination of normal/small TL ratio in patch and distal normal oral mucosa, we segregated OL patients in four subgroups (Table 3). This grouping will help to understand molecular pathogenesis and progression of precancerous conditions. Normal TL in patches against a background of short TL in normal oral mucosa, as defined in group B, might represent activation or increase in a TL maintenance mechanism at the site of a patch. Persistent reactivation might be more alarming in terms of risk of developing carcinoma. Long TL in patches could be indicative of greater survival and proliferating capacity against a background of oral mucosa with severely short TL. Interestingly some earlier studies have shown poor prognosis in the patients with a higher tumor to adjacent normal tissue TL ratio in oral and colorectal cancers [24, 42]. There is evidence that normal cells with short telomeres secrete signaling molecules which contribute to chromosomal instability (CIN) and disease progression in neighboring cells [56, 57, 58]. This suggests that identification of this group with close follow‐up prior to clinical tumor formation may reduce incidence of more fatal forms of oral malignancy. Evaluation of high‐risk presentations of OL (i.e. nonhomogeneous OL and dysplasia/carcinoma) as a single category demonstrated that it is associated with a small TL ratio in OLN. As expected, the smallest TL ratio was observed in the dysplasia/carcinoma group, followed by nonhomogeneous and homogeneous without dysplasia group in both OLN and OLP samples. These observations also provide validation of our approach of measuring the TL ratio.

Carrying out a follow‐up program accommodating a large number of OL cases for early detection of malignant transformation is a huge burden on health systems. Therefore, the identification of a potential high‐risk OL group is essential. We propose that in initial screening of OL patients using our approach, those with a smaller TL ratio in OLN (group B/C) could be categorized as high‐risk and considered for confirmatory biopsy and short‐interval follow‐up. Patients with normal OLN (group A/D) can be categorized as low‐risk and assigned to longer interval follow‐up. During the follow‐up period, if the low‐risk patients develop short OLN (B/C), they can be treated and referred to follow‐up as high risk. Transformation of OL into group B (short OLN, normal OLP) from any other TL group during the follow‐up could be considered alarming. More aggressive investigation and close surveillance may help diagnose early‐stage oral cancer.

However, we would like to caution that even this approach (of using PBMC as reference) may not provide normalization under certain situations. For example, in individuals aged less than 19 years, the TL among different tissues could be constantly evolving [45]. There could also be conditions which accelerate TL attrition in PBMC [59, 60]. These situations will have to be considered before using this approach. However, since it is not possible to find matched individual control for every variable, our approach of using paired PBMC samples as a reference provides the best possible solution and our data provide preliminary evidence in this regard. The method could also be applied to other precancerous conditions (such as in esophagus, cervix and others) and other cancers as well as for analyzing sperm telomere attrition in cases of infertility.

## 5 Conclusion

Relative TL of oral mucosa normalized to paired PBMC sample can serve as a standalone and universally comparable clinical marker of oral health. However, larger multicentric study is required for further validation of the approach.

## Conflict of interest

The authors declare no conflict of interest.

### Peer review

The peer review history for this article is available at https://publons.com/publon/10.1002/1878‐0261.13133.

## Author contributions

JP: envisioned the study, study design and supervision, method development, experimentation including qPCR, data analysis and interpretation, manuscript preparation. YR: clinical data collection, data curation, performing experiments, data analysis. SS, RG: cytological slide review. VM, PPR, AC, SSM, HB: patient selection. PKP, VC: provided resources, multidisciplinary coordination, intellectual discussion. PKP, VC, MAS, YR, PKP, VM: final editing of the manuscript; MAS: provided expert advice, assisted in data interpretation and critical review of the manuscript.

## Supporting information


**Table** **S1**. Age distribution among oral leukoplakia patients and healthy controls.
**Table** **S2**. Correlation of telomere length with duration high‐risk habit.
**Table** **S3**. Profile of high‐risk habit groups in healthy control and oral leukoplakia patients irrespective of other associated habits.
**Table** **S4**. Test of normality of the samples using Kolmogorov‐Smirnov Test of Normality.
**Table** **S5**. Correlation statistics of telomere length ratio of patches and paired distal normal site in oral leukoplakia patients.
**Table** **S6**. Significance of mean difference of telomere length ratio in oral leukoplakia patents and healthy controls with high‐risk habit.
**Table** **S7**. Profile of telomere length ratio in each high‐risk habit group of oral leukoplakia without other associated habits.
**Fig**. **S1**. Comparison rTL (TL ratio) measurements of oral mucosa using paired PBMC vs. external reference DNA.

## Data Availability

The supporting data are available in the supplementary material of this article (Fig. S1 and Table S1–S7).
