# Supplementary material for: A standalone approach to utilize telomere length measurement as a surveillance tool in oral leukoplakia
Source: Mol Oncol. 2022 Jan 7;16(8):1650–60. doi: 10.1002/1878-0261.13133 (PMC9019888; doi:10.1002/1878-0261.13133)
Supplement: Supplementary file 1 — Table S1. Age distribution among oral leukoplakia patients and healthy controls. Table S2. Correlation of telomere length with duration high‐risk habit. Table S3. Profile of high‐risk habit groups in healthy control and oral leukoplakia patients irrespective of other associated habits. Table S4. Test of normality of the samples using Kolmogorov‐Smirnov Test of Normality. Table S5. Correlation statistics of telomere length ratio of patches and paired distal normal site in oral leukoplakia patients. Table S6. Significance of mean difference of telomere length ratio in oral leukoplakia patents and healthy controls with high‐risk habit. Table S7. Profile of telomere length ratio in each high‐risk habit group of oral leukoplakia without other associated habits. Fig. S1. Comparison rTL (TL ratio) measurements of oral mucosa using paired PBMC vs. external reference DNA. [file MOL2-16-1650-s001.docx]

**Title: A standalone approach to utilize telomere length measurement as a surveillance tool in oral leukoplakia**

Jagannath Pal^1^, Yogita Rajput^1^, Shruti Shrivastava^2,3^, Renuka Gahine^1,3^, Varsha Mungutwar^4^,Tripti Barardiya^5^, Ankur Chandrakar^4^, Pinaka Pani Ramakrishna^6^, Sovna Shivani Mishra^6^,Hansa Banjara^4^, Vivek Choudhary^7^, Pradeep K. Patra^5^and Masood A. Shammas^8^

^1^ Multi-Disciplinary Research Unit (MRU), Pt. J.N.M. Medical College, Raipur, Chhattisgarh, India, ^2^Department of Pathology, Govt. Medical College, Rajnandgaon, Chhattisgarh, India, ^3^Department of Pathology, Pt. J.N.M. Medical College, Raipur, Chhattisgarh, ^4^Department of ENT, Dr. B.R.A.M. Hospital & Pt. J.N.M. Medical College, Raipur, Chhattisgarh, India, ^5^Department of Biochemistry, Pt. J.N.M. Medical College, Raipur, Chhattisgarh, India,^6^Govt. Dental College, Raipur, Chhattisgarh, India, ^7^Regional Cancer Centre, Dr. B.R.A.M. Hospital, Raipur, Chhattisgarh, India, ^8^Harvard (Dana Farber) Cancer Institute and VA Boston Healthcare System, Boston, USA

**Short title:** Telomere length in oral leukoplakia

**Correspondence:**

Jagannath Pal

Multi-Disciplinary Research Unit (MRU),

Pt. J.N.M. Medical College,

Raipur, Chhattisgarh, 492001

INDIA

E-mail: [jagannathpall@gmail.com](mailto:jagannathpall@gmail.com)

Tel.: +91-9903602662

**Supplementary Material:**

**Table S1. Age distribution among oral leukoplakia patients and healthy controls.**

| **Sample category (n)** | | | **Age (Y) Mean±SD** | |
| --- | --- | --- | --- | --- |
| **OL** | **Total (50)** | **Habit (47)** | 42.24±12.08 | 43.08±11.88 |
|  |  | **No habit (3)** |  | 29±7 |
| **HC** | **Total (44)** | **Habit (19)** | 36.36±13.47 | 34.36±7.58 |
|  |  | **No habit (25)** |  | 37.88± 16.63 |

OL: Oral leukoplakia;Y: In years; HC: Healthy controls; Habit: Oral high-risk habit; No habit: No oral high-risk habit; n: number of subjects.

**Table S**2.**Correlation of telomere length with duration of high-risk habits.**

| **Samples** | **Duration of habit (Y)**  **Mean ±SD**  **(Range)** | **Correlation**  **Duration of habit vs TL : R(p)** | **Correlation**  **Duration of habit vs age : R(p)** |
| --- | --- | --- | --- |
| **OL (oral Habit)** | 15.47±11.40  (2-50) | OLN: 0.114 (0.449)  OLP: -0.0728 (0.634) | 0.6217 (< 0.00001) |
| **HC (oral Habit)** | 10.13 ± 8.343  (2-30) | 0.0177 (0.943) | 0.583 ( 0.0877 ) |

TL: Telomere Length;Y: In years; OL: Oral Leukoplakia patients; HC: Healthy controls; OLN: Paired distal normal mucosa in OL patients; OLP: Oral patch in OL patients; R: The *r* statistics (measure of correlation); *p: p value is significant at the significance level p=0.05.

**Table S3**. **Profile of high-risk habit groups in HC and OL irrespective of other associated habits.**

| **High-risk habits** | **HC with High-risk habits (19)** | **OL with High-risk habits (47)** |
| --- | --- | --- |
|  | **%(n)**  **(irrespective of associated habits)** | **%(n)**  **(irrespective of associated habits)** |
|  |  |  |
| **Smoking** | 37(6) | 38(18) |
| **Chewing Raw Tobacco (Khaini)** | 37(7) | 44(21) |
| **Chewing Gutkha** | 53(10) | 55(26) |
| **Drinking Alcohol** | 47(9) | 42(20) |

OL: Oral Leukoplakia patients; HC: Healthy control; n: number of subjects having the high-risk habit (irrespective of any other habits).

**Table S4. Test of normality of the samples using the Kolmogorov-Smirnov test of normality.**

|  | **HC** | | | **OL** | | | | | |
| --- | --- | --- | --- | --- | --- | --- | --- | --- | --- |
|  | **Total(44)** | **NOHC(25)** | **OHC (19)** | **Total (50)** | | **High risk oral habit(47)** | | **No high-risk habit (3)** | |
|  |  |  |  | **OLN** | **OLP** | **OLN** | **OLP** | **OLN** | **OLP** |
| **K-S test statistic (D)** | 0.096 | 0.141 | 0.221 | 0.090 | 0.091 | 0.110 | 0.095 | NA | NA |
| **P** | 0.777 | 0.653 | 0.27 | 0.780 | 0.766 | 0.580 | 0.757 | NA | NA |
| **Normality** | Yes | Yes | Yes | Yes | Yes | Yes | Yes |  |  |

HC: Healthy controls; OL: Oral leukoplakia; NOHC: Healthy controls without oral habit; OHC: Healthy controls with high-risk oral habit; OLN: Paired distal normal mucosa in OL patients; OLP: Oral patch in OL patients; NA: Not applicable.

**Table S5**. **Correlation statistics of TL ratio of patches (P) and paired distal normal site(N) in OL patients**

| **OL patients** | **Correlation (R)** | | |
| --- | --- | --- | --- |
|  | **Age vs OLN**  **R(p)** | **Age vs OLP**  **R(p)** | **OLN vs OLP**  **R(p)** |
| **Total (50)** | 0.160 (0.268) | 0.090 (0.539) | 0.389(0.005*) |
| **Habit (47)** | 0.141(0.343) | 0.037  (0.804) | 0.381  (0.008*) |
| **No hobbit (3)** | NA | NA | NA |

OLN: Paired distal normal mucosa in OL patients; OLP: Oral patch in OL patients; R: The *r* statistics (measure of correlation); *p: p value is significant at the significance level p=0.05; NA: Not applicable.

**Table S6. Significance of mean difference of TL ratio in OL patients and HC with high-risk habit**

| **OL Patients (n)** | n | **TL ratio OL vs habit HC** | |
| --- | --- | --- | --- |
|  |  | **OLN** | **OLP** |
|  |  | **p** | **P** |
| **Total** | 50 | 0.031* | 0.203 |
| **Habit** | 47 | 0.037* | 0.294 |
| **No habit** | 3 | 0.26 | 0.159 |

OLN: Paired distal normal mucosa in OL patients; OLP: Oral patch in OL patients; Habit: Oral high-risk habit, No habit: No oral high-risk habit, n: Number of subjects, p : Significance of mean difference compared to high-risk oral habit healthy control (HC), * p value is significant at the significance level p≤0.05

**Table S7. Profile of TL ratio in each high-risk habit groups of OL without other associated habits**

| **High-risk habits** | **OL with High-risk habits (47)** | | |
| --- | --- | --- | --- |
|  | **%(n)**  **(no other associated habits)** | **TLratio : OLN** | **TL ratio: OLP** |
|  |  | **Mean ± SD**  **(p)** | **Mean ± SD**  **(p)** |
| **Smoking** | 15(7) | 0.89±0.38  (0.010*) | 0.83±0.34  (0.003*) |
| **Chewing Raw Tobacco (Khaini)** | 10(5) | 0.68±0.30  (0.0004*) | 0.89±0.37  (0.021*) |
| **Chewing Gutkha** | 23(11) | 0.82±0.20  (0.0001*) | 1.01±0.24  (0.022*) |
| **Drinking Alcohol** | 0(0) | NA | NA |

OLN: Paired distal normal mucosa in OL patients; OLP: Oral patch in OL patients; n: Number of subjects having the high-risk habit (no other habits), p : Significance of mean difference compared to no high-risk oral habit healthy control (HC), * p value is significant at the significance level p≤0.05, NA: Not applicable.

**
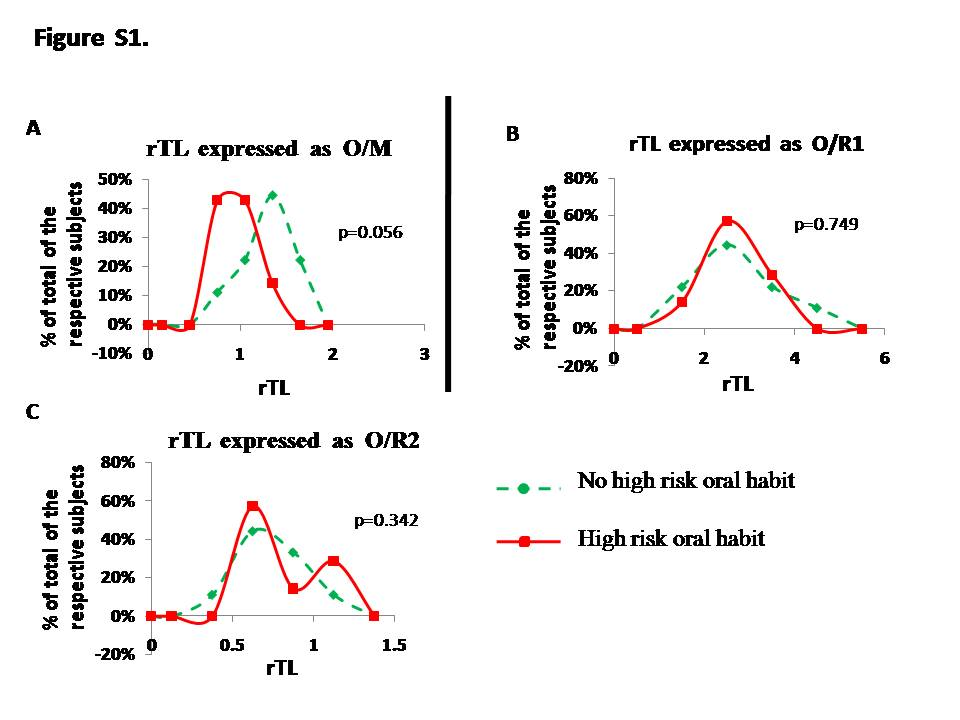
**

**Figure S1. Comparison rTL (TL ratio) measurements of oral mucosa using paired PBMC vs. external reference DNA.** Relative telomere length (rTL) was measured in healthy control subjects with (n=7) and without (n=9) high-risk oral habits using PBMC or reference DNA as normalization control as indicated**.** (**A**) Frequency distribution of rTL using paired PBMC DNA as normalizing control (O/M ratio). (**B**) Frequency distribution of rTL using HCT116 cell line DNA as normalizing reference DNA (O/R1 ratio).(**C**) Frequency distribution of of rTL using pooled DNA as normalizing reference DNA (O/R2 ratio). For each batch of qPCR run, along with the oral samples, paired PBMC and the DNA references were run in triplicate simultaneously. The T/S value of the reference DNA was used only for the corresponding batch of the samples run on the same plate. PBMC: Peripheral Blood Mononuclear Cells, O: T/S of oral mucosa; M: T/S of paired PBMC of the same subject; R1: T/S of HCT116 cell line; R2: T/S of pooled DNA from 10 different oral brush biopsy samples of healthy control; T/S: Telomere sequence amplification/ single copy geneamplification [(2^-ΔCt^, whereas ΔCt = Ct (telomere) – Ct (36B4)],Significance of difference(p<0.05) between oral habit and no habit groups were determined by Mann-Whitney U test.
